# Selective progesterone receptor modulator (SPRM) ulipristal acetate (UPA) and its effects on the human endometrium

**DOI:** 10.1093/humrep/dew359

**Published:** 2017-01-27

**Authors:** L.H.R. Whitaker, A.A. Murray, R. Matthews, G. Shaw, A.R.W. Williams, P.T.K. Saunders, H.O.D. Critchley

**Affiliations:** 1 MRC Centre for Reproductive Health, The University of Edinburgh, The Queen's Medical Research Institute, 47 Little France Crescent, Edinburgh EH16 4TJ, UK; 2 Division of Pathology, The University of Edinburgh, The Royal Infirmary of Edinburgh, 51 Little France Crescent, Edinburgh EH16 4SA, UK; 3 MRC Centre for Inflammation Research, The University of Edinburgh, The Queen's Medical Research Institute, 47 Little France Crescent, Edinburgh EH16 4TJ, UK

**Keywords:** selective progesterone receptor modulator (SPRM), endometrium, proliferation, androgen receptor, progesterone receptor, estrogen receptor, progesterone-regulated genes

## Abstract

**STUDY QUESTION:**

What is the impact of administration of the selective progesterone receptor modulator (SPRM), ulipristal acetate (UPA) on the endometrium of women with fibroids?

**SUMMARY ANSWER:**

UPA administration altered expression of sex-steroid receptors and progesterone-regulated genes and was associated with low levels of glandular and stromal cell proliferation.

**WHAT IS KNOWN ALREADY:**

Administration of all SPRM class members results in PAEC (progesterone receptor modulator associated endometrial changes). Data on the impact of the SPRM UPA administration on endometrial sex-steroid receptor expression, progesterone (P)-regulated genes and cell proliferation are currently lacking.

**STUDY DESIGN SIZE, DURATION:**

Observational study with histological and molecular analyses to delineate impact of treatment with UPA on endometrium. Endometrial samples (*n* = 9) were collected at hysterectomy from women aged 39 to 49 with uterine fibroids treated with UPA (oral 5 mg daily) for 9–12 weeks. Control proliferative (*n* = 9) and secretory (*n* = 9) endometrium from women aged 38–52 with fibroids were derived from institutional tissue archives.

**PARTICIPANTS/MATERIALS, SETTING, METHODS:**

Study setting was a University Research Institute. Endometrial biopsies were collected with institutional ethical approval and written informed consent. Concentrations of mRNAs encoded by steroid receptors, P-regulated genes and factors in decidualised endometrium were quantified with qRT-PCR. Immunohistochemistry was employed for localization of progesterone (PR, PRB), androgen (AR), estrogen (ERα) receptors and expression of FOXO1, HAND2, HOXA10, PTEN homologue. Endometrial glandular and stromal cell proliferation was objectively quantified using Ki67.

**MAIN RESULTS AND THE ROLE OF CHANCE:**

UPA induced morphological changes in endometrial tissue consistent with PAEC. A striking change in expression patterns of PR and AR was detected compared with either proliferative or secretory phase samples. There were significant changes in pattern of expression of mRNAs encoded by *IGFBP-1, FOXO1, IL-15, HAND2, IHH* and *HOXA10* compared with secretory phase samples consistent with low agonist activity in endometrium. Expression of mRNA encoded by *FOXM1*, a transcription factor implicated in cell cycle progression, was low in UPA-treated samples. Cell proliferation (Ki67 positive nuclei) was lower in samples from women treated with UPA compared with those in the proliferative phase.

**LARGE SCALE DATA:**

N/A.

**LIMITATIONS REASONS FOR CAUTION:**

A small number of well-characterized patients were studied in-depth. The impacts on morphology, molecular and cellular changes with SPRM, UPA administration on symptom control remains to be determined.

**WIDER IMPLICATIONS OF THE FINDINGS:**

P plays a pivotal role in endometrial function. P-action is mediated through interaction with the PR. These data provide support for onward development of the SPRM class of compounds as effective long-term medical therapy for heavy menstrual bleeding.

**STUDY FUNDING/COMPETING INTEREST(S):**

H.O.D.C. received has clinical research support for laboratory consumables and staff from Bayer Pharma Ag and provides consultancy advice (no personal remuneration) for Bayer Pharma Ag, PregLem SA, Gedeon Richter, Vifor Pharma UK Ltd, AbbVie Inc.; A.R.W.W. has received consultancy payments from Bayer, Gedeon Richter, Preglem SA, HRA Pharma; L.H.R.W., A.A.M., R.M., G.S. and P.T.K.S. have no conflicts of interest. Study funded in part from each of: Medical Research Council (G1002033; G1100356/1; MR/N022556/1); National Health Institute for Health Research (12/206/520) and TENOVUS Scotland.

## Introduction

Heavy menstrual bleeding (HMB) is a common condition affecting up to one in four women of reproductive age (Shapley *et al*., 2004). This condition has significant impact upon the physical, social, emotional and material quality of life of women (NICE, 2007). In the USA, conservative estimates of annual direct and indirect costs are $1 and $12 billion, respectively (Liu *et al*., 2007). In the UK, it is estimated that 1 million women annually seek help for HMB (NICE, 2007). There are multiple etiologies that are associated with HMB including fibroids (leiomyomas) (Munro *et al*., 2011). In women with symptomatic fibroids, HMB is the major complaint for which treatment is sought and is a leading indication of hysterectomy in pre-menopausal women (Merrill, 2008) resulting in sterility, an unacceptable side effect for many women. The mechanisms responsible for the development of these benign tumours is not fully understood but it is well established that fibroid growth is regulated by the actions of the sex-steroids estrogen (E) and progesterone (P) (Yin *et al*., 2007, 2010; Bulun, 2013).

Progesterone is a critical regulator of female reproduction and plays a pivotal role in endometrial differentiation and its withdrawal following demise of the corpus luteum during a non-pregnant cycle precipitates endometrial shedding during menstruation (Maybin and Critchley, 2015). Progesterone responses are mediated through interaction of the steroid with the progesterone receptor (PR); a ligand-activated transcription factor. It has been demonstrated that two main isoforms of the receptor (termed PR-A and PR-B) are present within the endometrium (Patel *et al*., 2015). Expression and relative abundance of these isoforms within the tissue contribute to normal uterine function and, if dysregulated, uterine pathophysiology. Notably, activation of P-responsive genes has been linked with reduced apoptosis and enhanced proliferation of fibroid cells (Yin *et al*., 2007, 2010).

Administration of synthetic progestins is a medical therapy currently used for managing HMB, however, these therapies often either fail to fully resolve symptoms or are associated with unacceptable side effects (Roberts *et al*., 2011). The development of a new class of synthetic compounds the ‘selective progesterone receptor modulators’ (SPRMs) exhibiting mixed agonist and antagonist activity appears to offer a new approach to medical management of hormone dependent uterine disorders such as HMB and fibroids (Wagenfeld *et al*., 2016). Class members include mifepristone, asoprisnil and ulipristal acetate (UPA).

Administration of the SPRMs mifepristone and asoprisnil has been reported to reduce both fibroid volume and menstrual blood loss (Wilkens *et al*., 2008; Engman *et al*., 2009). Currently the SPRM, UPA is the only member of this class of drug licensed in Europe for short-term treatment of fibroids prior to hysterectomy and more recently (in Europe) for intermittent treatment of moderate to severe symptoms of uterine fibroids in adult women of reproductive age. In clinical studies, utilizing a regime of repeated doses of UPA it has been shown that median fibroid volume was reduced by 45% of women receiving treatment and nearly 90% of patients reported a significant reduction in menstrual bleeding (Donnez *et al*., 2014).

UPA administration, in common with that of other SPRMs, is associated with morphological changes of the endometrium. Characteristics of these effects include large cystic glands, changes within the stromal compartment including the fibroblasts and vasculature and are now recognized as a distinct histological entity, termed progesterone receptor modulator associated endometrial changes (PAEC) (Williams *et al*., 2012).

Studies have demonstrated that the SPRMs mifepristone and UPA both exert anti-proliferative and pro-apoptotic effects on leiomyoma cells *in vitro* (Luo *et al*., 2010; Yin *et al*., 2010), which may explain mechanisms by which fibroid volume is reduced but there are very limited data as to the mechanism of SPRM action within the intact endometrium.

The mechanism by which control of bleeding is achieved is unclear. Clinical trials report bleeding control in up to 98% of women but only around 70–74% exhibit PAEC (Donnez *et al*., 2012a,b; Williams *et al*., 2012). Furthermore, some patients who continue to ovulate still achieve amenorrhoea (Chabbert-Buffet *et al*., 2007). Hence it is essential to delineate potential endometrial causes for the mitigation of menstrual bleeding.

In this study, we have used histological and molecular analyses to delineate the impact of treatment with UPA for 9–12 weeks on endometrial tissue focusing on expression of sex-steroid receptors, the products of genes known to be progesterone-regulated during the normal cycle and the impact upon endometrial cell proliferation.

The data presented here offer evidence that UPA, a licensed SPRM alters expression of sex-steroid receptors and PR regulated genes without increasing endometrial cell proliferation.

## Materials and Methods

### Patients and samples

After ethical approval (Research Ethics Committee 12/SS/0238; 10/S1402/59) and written informed consent, endometrial biopsies (*n* = 9) were collected at the time of surgery from women aged 39 to 49 years with symptomatic uterine fibroids treated with UPA (oral 5 mg daily) for 9–12 weeks (63–84 days) up to the time of hysterectomy (Table I). All subjects with one exception described excellent control of bleeding whilst receiving UPA with either complete amenorrhoea or only occasional spotting. Control proliferative (*n* = 9) and secretory (*n* = 9) endometrium from pre-menopausal women aged 38–52 undergoing hysterectomy for symptomatic fibroids were utilized from tissue archives (REC: 10/S1402/59). All control patients were women with fibroids who reported regular menstrual cycles and no preoperative hormone use. Median estradiol and progesterone serum levels were 497 (range 12.5–1272) and <3 (<3–5.7) in the proliferative group and 323 (range 77.4–530) and 27.1 (14.8–45.7) in the secretory group. Age and BMI were not significantly different from the UPA-treated group. Control endometrial tissues were staged based on standard histological criteria (Noyes *et al*., 1950), the patient's reported last menstrual period, and circulating estradiol and progesterone levels at the time of collection.

**Table I:** Subject characteristics and endometrial histology.

Subject	Age (Year)	Symptoms	Submucosal component of fibroid	Duration of UPA (days)	Bleeding control	Histology
A	43	HMB pain	No	83	Amenorrhoea	Extensive features of PAEC with widespread cystic glandular dilatation. Occasional thick-walled vessels
B	48	HMB pain	No	63	Amenorrhoea	Extensive features of PAEC with widespread cystic glandular dilatation. No abnormal vessels
C	39	HMB	Yes	84	Amenorrhoea	Extensive features of PAEC with widespread cystic glandular dilatation. No abnormal vessels
D	39	HMB pressure	Yes	76	Occasional spotting only	Some features of PAEC with occasional dilated cystic glands with tortuous morphology
E	49	HMB pressure	No	77	Amenorrhoea	Some features of PAEC with occasional dilated cystic glands with tortuous morphology
F	45	HMB	Yes	81	Increased HMB	Some features of PAEC with occasional dilated cystic glands with tortuous morphology
G	45	HMB pressure	Yes	84	Amenorrhoea	Some features of PAEC with occasional dilated cystic glands with tortuous morphology. Several thick-walled vessels
H	47	HMB pressure	Yes	75	Irregular spotting	Some features of PAEC with occasional dilated cystic glands with tortuous morphology
I	46	HMB pressure	No	63	Amenorrhoea	Minimal features of PAEC with occasional partially dilated cystic glands

UPA, ulipristal acetate; HMB, heavy menstrual bleeding; PAEC, progesterone receptor modulator associated endometrial changes.

### Gene expression analyses

RNA was isolated from endometrial samples using Qiagen RNAeasy mini kit as per manufacturer's protocol (Qiagen, UK). Quality of RNA was analyzed using the Agilent RNA 600 nano kit according to manufacturer's protocol (Agilent Technologies, USA). RNA integrity numbers were all greater than 7.7. cDNA was prepared using Superscript Vilo cDNA kit (Invitrogen, UK) using 100 ng RNA as template. Taqman RT-qPCR was performed in triplicate reactions using primers designed using the online Universal Probe Assay (Roche Diagnostics, USA) and synthesized by Eurofins Genomics (Germany) (Table II) on an ABI Prism Cycler (Applied Biosystems USA). Indian hedgehog (*IHH*) and phosphatase and tensin homologue (*PTEN*) primers were bought as pre-validated sets (Applied Biosystems, UK) and used according to the manufacturer's protocol. Relative quantification of target genes were analyzed after normalization to the geometric mean of endogenous ATP synthase H+ transporting mitochondrial F1 complex, Beta polypeptide (*ATPB5*) and *18s* and a control sample by the comparative ΔΔCT method.

**Table II:** Primers and probes for quantitative PCR.

**Target gene**	**Forward primer**	**Reverse primer**	**Roche probe**
*PR*	tttaagagggcaatggaagg	cggattttatcaacgatgcag	11
*PRB*	aatgggctgtaccgagaggt	tctcagtccctcgctgagtt	45
*AR*	gctgatcataggcctctctc	tgccctgaaagcagtcctct	14
*ESR1*	aaccagtgcaccattgataaaa	tcctcttcggtcttttcgtatc	68
*IGFBP-1*	aatggattttatcacagcagacag	ggtagacgcaccagcagagt	58
*FOXO1*	aagggtgacagcaacagctc	ttccttcattctgcacacga	11
*IL-15*	cagatagccagcccatacaag	ggctatggcaaggggttt	46
*HAND2*	tcaagaagaccgacgtgaaa	gttgctgctcactgtgcttt	35
*HOXA10*	ccttccgagagcagcaaa	ttggctgcgttttcacct	61
*FOXM1*	actttaagcacattgccaagc	cgtgcagggaaaggttgt	11
*COUP TFII*	ccatagtcctgttcacctcaga	aatctcgtcggctggttg	36
*BMP2*	cggactgcggtctcctaa	ggaagcagcaacgctagaag	49

### Immunohistochemistry

Tissue samples were fixed in 4% neutral buffered formalin, sectioned, processed and stained with Haematoxylin and Eosin by standard methods. For immunohistochemistry 5 μm sections were subjected to antigen retrieval (Table III); non-specific activity was blocked sequentially with 3% hydrogen peroxide and appropriate serum before overnight incubation at 4°C with antibodies specific to: PR, PRB, androgen receptor (AR), estrogen receptor alpha (ERα), FOXO1, HAND2, HOXA10, PTEN and Ki67 (Table III). Appropriate matched IgG was applied as a negative control. Sections were incubated with ImmPRESS™ Ig reagents (Table III); bound antibodies were visualized using 3,3′-diaminobenzidine (Vector Laboratories, UK). Sections were counterstained with haemotoxylin and mounted in Pertex (Cellpath Technologies, UK). Representative images were captured using an Olympus BX51 microscope equipped with a Nikon DSFi1 camera (Olympus Optical Co. and Nikon Ltd, UK).

**Table III:** Antibodies for immunohistochemistry.

Protein	Supplier	Reference	Antibody type	Host	Dilution (normal horse serum)	Retrieval Buffer	ImmPRESS™ kit
PR	Dako	A0098	Polyclonal	Rabbit	1:200	Citrate	Rabbit MP-7401
PRB	Cell signalling	3157S	Monoclonal	Rabbit	1:800	Citrate	Rabbit MP-7401
AR	Spring bioscientific	M4070	Monoclonal	Rabbit	1:200	Citrate	Rabbit MP-7401
ERα	Vector	VP-E614	Monoclonal	Mouse	1:5000	Citrate	Mouse MP-7402
FOXO1	Cell signalling	2880	Monoclonal	Rabbit	1:250	Citrate	Rabbit MP-7401
PTEN	Dako	M3627	Monoclonal	Mouse	1:750	Citrate	Mouse MP-7402
HAND2	Santa Cruz	SC9409	Polyclonal	Goat	1:200	Citrate	Goat MP-7405
HOXA10	Santa Cruz	SC17159	Polyclonal	Goat	1:200	Citrate	Goat MP-7405
Ki67	NovaCastra	NCL-Ki67-MM1	Monoclonal	Mouse	1:500	TRIS	Mouse MP-7402

### Analysis and scoring of proliferation marker expression

Ki67 stained sections (*n* = 6/group) were scanned using a Zeiss Imager A1microscope, fitted with a Prior Proscan II automatic stage (Zeiss, UK). For each scanned image 20 random fields were automatically selected by the programme and a 28 × 20 point grid was applied using Image Pro Plus 7.0 software. There were a total of 560 points (intersections of the grid) per field categorized as: (i) stained (positive) stromal cells, (ii) stained (positive) glandular cells, (iii) unstained (negative) stromal cells, (iv) unstained (negative) glandular cells and (v) all other points not overlaying any cells (e.g. empty slide space, lumen, connective tissue or blood). Points not overlaying a cell were excluded from analysis. The proportion of tissue in which each of the categorized cell type occupied was expressed as a percentage of total calculated area; cell proliferation index.

### Statistics

Data were analyzed using Graphpad prism software (Graphpad, USA) utilizing non-parametric tests. Kruskal–Wallis test was used to determine differences between sample groups. Results are presented as mean ± SEM. *P* < 0.05 was considered to be statistically significant.

## Results

### Impact of UPA administration on endometrial morphology

**Figure 1:**
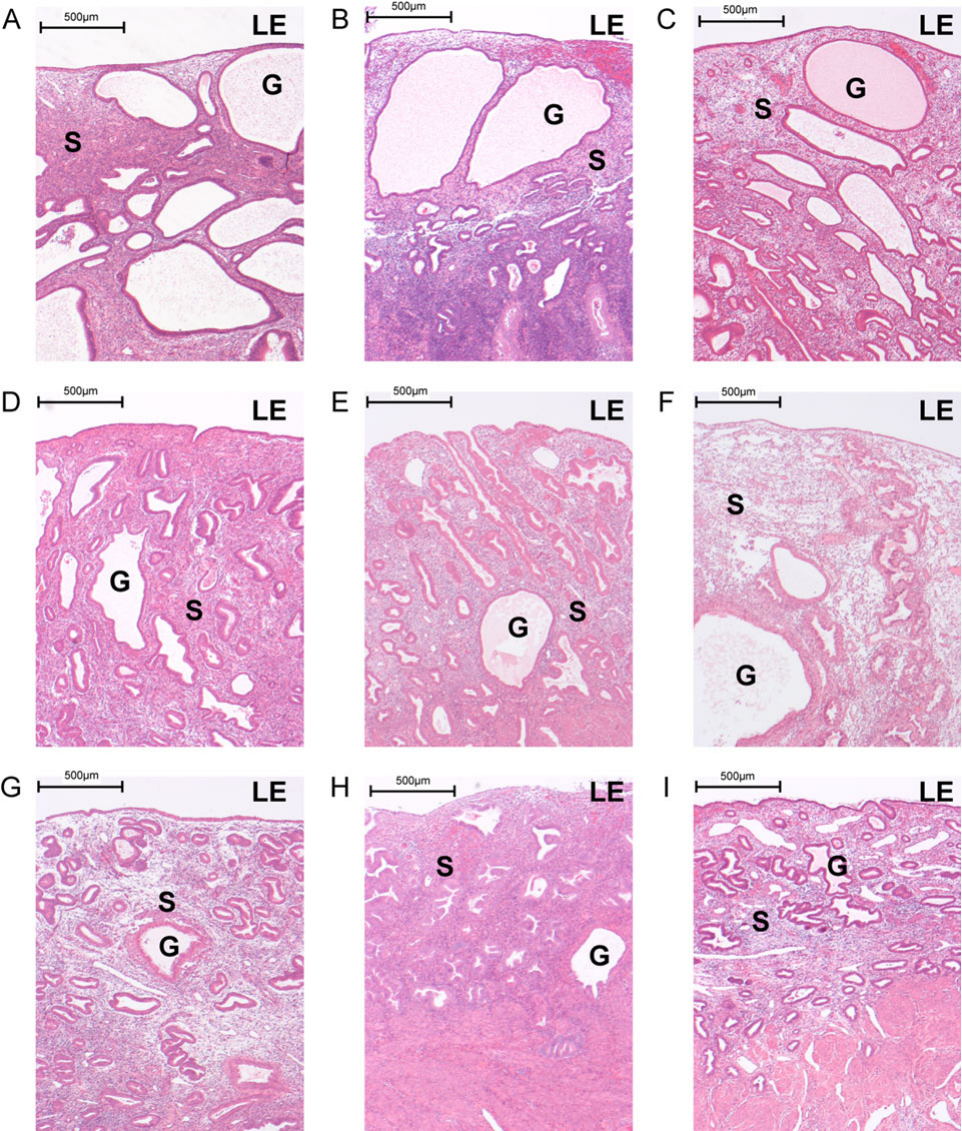
Selective progesterone receptor modulator (SPRM), ulipristal acetate (UPA) modifies endometrial morphology. Haematoxylin and eosin staining of full thickness endometrium from hysterectomy specimens of nine subjects with uterine fibroids exposed to UPA for up to 12 weeks. All specimens exhibited progesterone receptor modulator associated endometrial changes (PAEC) but with variation in the degree of cystic glandular dilatation, and in overall endometrial thickness. Subjects A–C showed widespread extensive cystic dilatation that was less frequently observed in subjects D–H. Subject I had minimal cystic change. No subjects exhibited histological evidence of inflammation, hyperplasia or neoplasia. ×4 magnification (scale bar = 500 µm); LE, Luminal epithelium; G, Glands; S, Stroma.

Endometrium from all women treated with UPA showed some histological features of PAEC (Table I). Individual images are illustrated in Fig. 1.  Some showed more extensive cystic glandular dilatation than others (Fig. 1A–C); the extent of dilatation did not correlate to the presence of submucosal fibroids, duration of treatment or control of bleeding (Table I). No subjects exhibited histological evidence of inflammation, hyperplasia or neoplasia.

### Treatment with UPA increases concentrations of mRNAs encoded by sex-steroid hormone receptors

**Figure 2:**
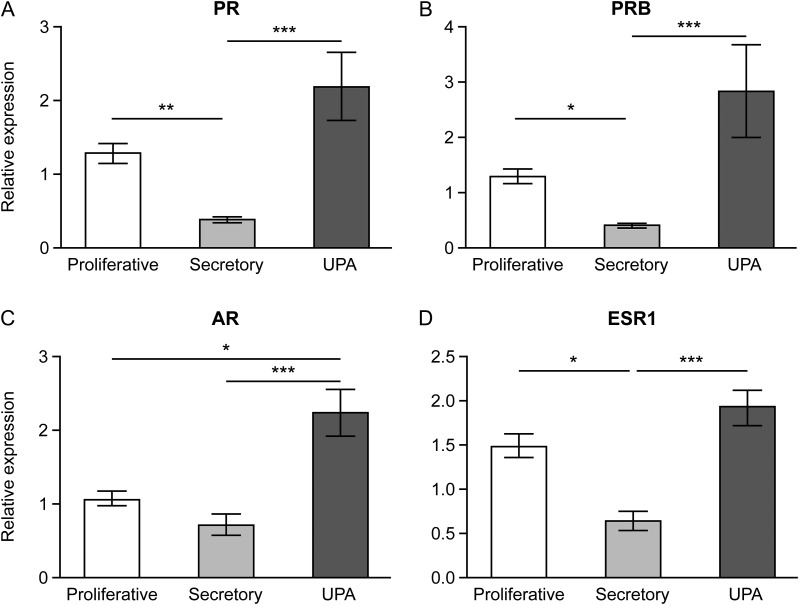
Treatment with SPRM, UPA, increased the concentration of mRNAs encoding sex-steroid receptors in tissue extracts from human endometrium as determined by qRT-PCR. Samples were from women with fibroids obtained during the proliferative and secretory phases or after UPA administration: *n* = 9 for each group. PR (**A**), PRB (**B**), AR (**C**), ESR1 (**D**). *P < 0.05, **P < 0.01, ***P < 0.001. Bars: mean ± SEM.

Concentrations of *PR* and *PRB* mRNAs were significantly lower in secretory endometrium compared to proliferative tissue. UPA administration was associated with significantly higher *PR* and *PRB* mRNA concentrations than secretory phase endometrium but was not significantly different to proliferative phase samples (Fig. 2A and B). Total *AR* mRNA levels in UPA-treated samples were significantly increased compared to both proliferative and secretory phase samples. *AR* mRNA concentrations were not significantly different between proliferative and secretory phase samples (Fig. 2C). Concentrations of *ESR1* (ERα) mRNA in secretory phase were significantly lower than proliferative phase and concentrations in UPA-treated samples were significantly higher than secretory phase. There was no significant different in *ESR1* concentration between proliferative phase and UPA-treated samples (Fig. 2D)..

### Immunoexpression of endometrial sex-steroid receptors was altered by UPA administration

**Figure 3:**
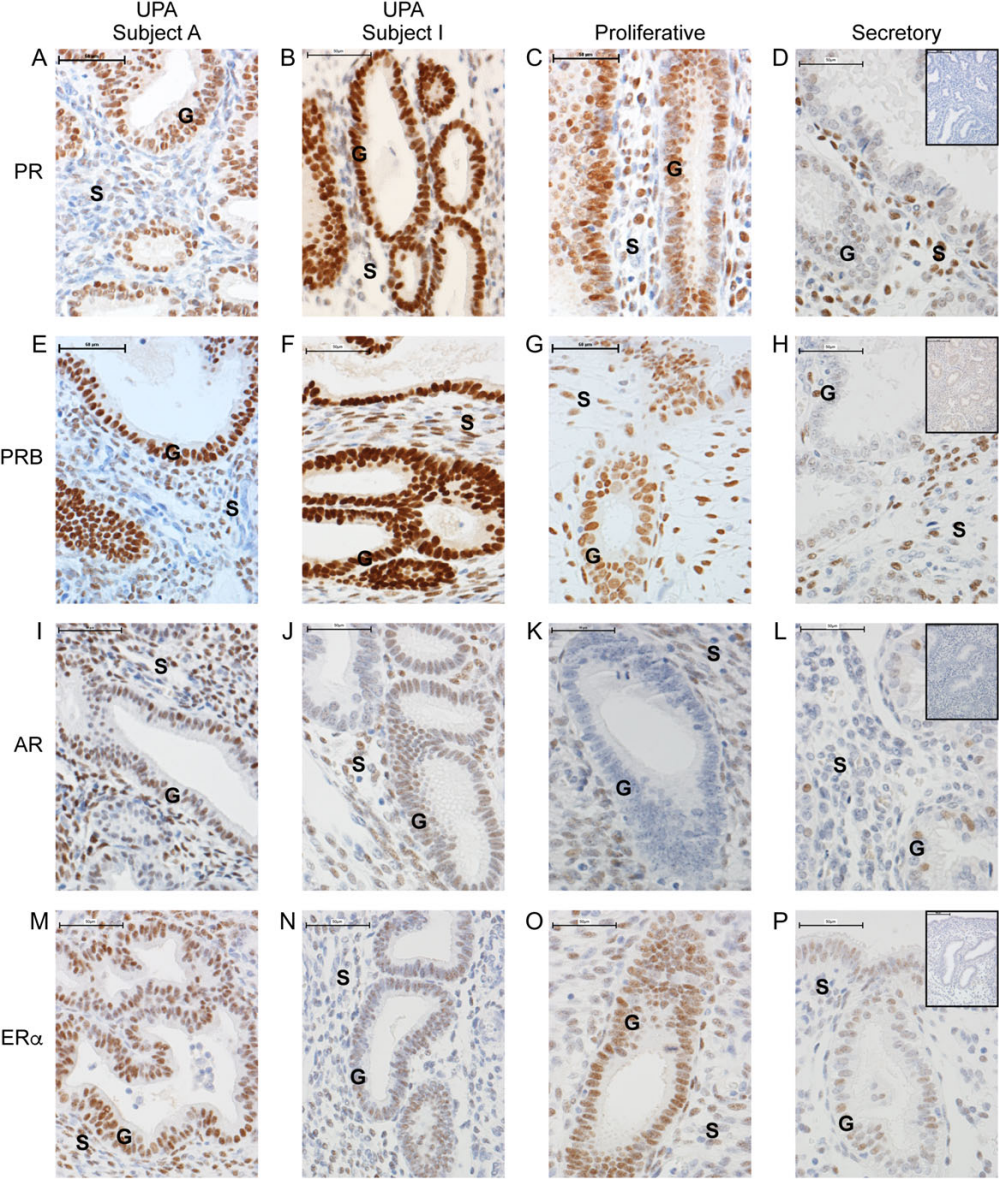
Administration of SPRM, UPA, modulates sex-steroid receptor localization. Representative immuno-localization of progesterone receptor (PR; **A**–**D**), PRB (**E**–**H**), androgen receptor (AR; **I**–**L**) and estrogen receptor alpha (ERα; **M**–**P**) in endometrium from woman with fibroids during proliferative and secretory stages and after UPA administration. Subject A shows endometrium in which PAEC are characterized by extensive cystic glandular dilatation; Subject I has PAEC with minimal cystic change. Neither subject had evidence of submucosal fibroids, and both women described amenorrhoea on UPA treatment. Samples from UPA-treated women displayed intense immunopositive (+ = positive and − = negative) glandular nuclei with only a few immunopositive cells in the stroma, a result in contrast with proliferative phase (G+S+) or secretory phase (G−S+).  ×40 magnification (scale bar = 50 µm); G, Glands; S, Stroma. Negative controls shown as inserts on secretory endometrium.

In agreement with previous studies (Lessey *et al*., 1988; Wang *et al*., 1998) intense immunopositive staining for PR (with antibody recognizing both isoforms) was detected in cell nuclei in both glandular epithelial cells and stromal fibroblasts in proliferative endometrium. Intensity was reduced in epithelial cell nuclei in the secretory phase (Fig. 3C versus D). UPA-treated endometrium with marked and minimal cystic glandular dilatation (Fig. 3A and B, respectively) showed a pattern of PR immunopositive staining characterized by intense staining of nuclei in glandular epithelium and weak/negligible immunoexpression in stromal fibroblasts. This pattern did not phenocopy either proliferative or secretory endometrium (Fig. 3C and D). These results were mirrored by results obtained using a PRB-specific antibody (Fig. 3E–H).

Consistent with previous findings in our group (Marshall *et al*., 2011), intense immunopositive staining for AR was detected in nuclei of stromal fibroblasts in proliferative endometrium (Fig. 3K); AR positive epithelial cells were only detected in secretory phase (Fig. 3L) coincident with a reduction in staining intensity in stromal cells. Immunostaining of UPA-treated endometrial sections revealed a unique pattern characterized by intense immunopositive staining of cell nuclei in both epithelial cells and stromal fibroblasts (Fig. 3I and J). The expression profile of ERα (*ESR1*) protein differed between proliferative and secretory endometrium with reduced immunoexpression detected in secretory phase tissue in both stromal fibroblasts and glandular epithelium (Fig. 3O and P). ERα immunoexpression in UPA-treated endometrium mirrored that of proliferative endometrium but was less intense in subjects with minimal cystic glandular dilatation (Fig. 3M and N).

### The impact of UPA administration on PR responsive genes

**Figure 4:**
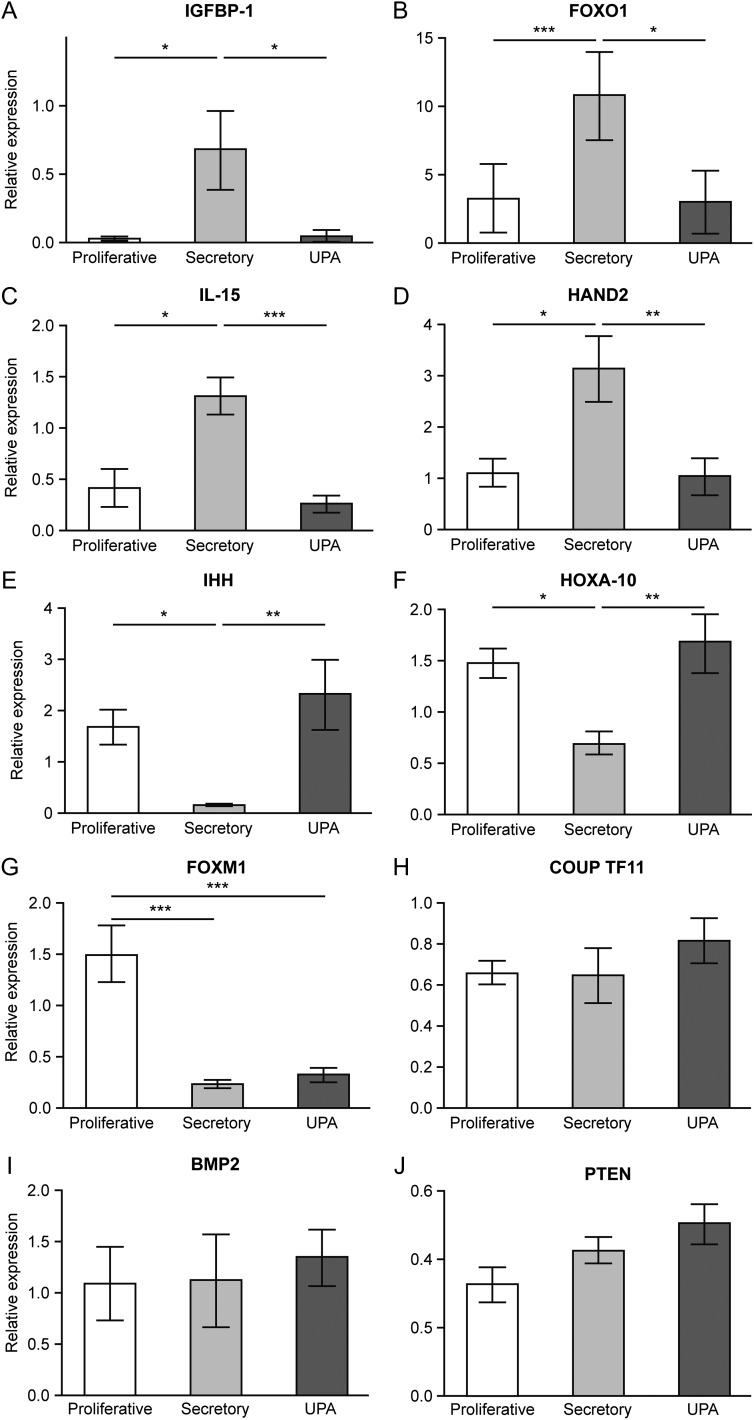
Selective progesterone (P) receptor modulator, UPA, administration impacts on concentrations of P-responsive genes. Relative quantification of P-responsive genes in tissue extracts from human endometrium as determined by qRT-PCR. Samples were from women with fibroids obtained during the proliferative and secretory phases or after UPA treatment: *n* = 9 for each group. IGFBP-1 (**A**), FOXO1 (**B**), IL-15 (**C**), HAND2 (**D**), IHH (**E**), HOXA-10 (**F**), FOXM1 (**G**), COUP TF11 (**H**), BMP2 (**I**), PTEN (**J**). *P < 0.05, **P < 0.01, ***P < 0.001. Bars: mean ± SEM.

As UPA is a SPRM, further investigations focused on genes known to be regulated during the progesterone-dominated secretory phase of the cycle and associated with decidualisation (Wetendorf and DeMayo, 2012). In contrast to total concentrations of mRNAs in tissues collected during the secretory phase three patterns were detected: a significant increase, a significant decrease and no change. Specifically, treatment with UPA resulted in a significant decrease in mRNAs encoded by *IGFBP-1* (Fig. 4A), *FOXO1* (Fig. 4B), *IL15* (Fig. 4C) and *HAND2* (Fig. 4D) compared with secretory phase endometrium. There was no significant difference in mRNA levels of *IGFBP-1, FOXO1, IL15* and *HAND2* between proliferative and UPA-treated endometrium. In contrast, endometrial mRNA concentrations of *IHH* and *HOXA10* (Fig. 4E and F) were significantly increased between samples from women treated with UPA and those samples collected in the secretory phase. There was no significant difference between proliferative phase and UPA-treated endometrium. Concentrations of *FOXM1* mRNA were significantly decreased in both secretory phase and UPA samples compared with proliferative phase (Fig. 4G). There was no significant menstrual cycle or UPA-dependent change in *COUP TFII, BMP2* or *PTEN* although a weak UPA effect was noted (Fig. 4H–J).

### Impact of UPA on immunoexpression of proteins normally present in the progesterone-dependent secretory phase of the cycle

**Figure 5:**
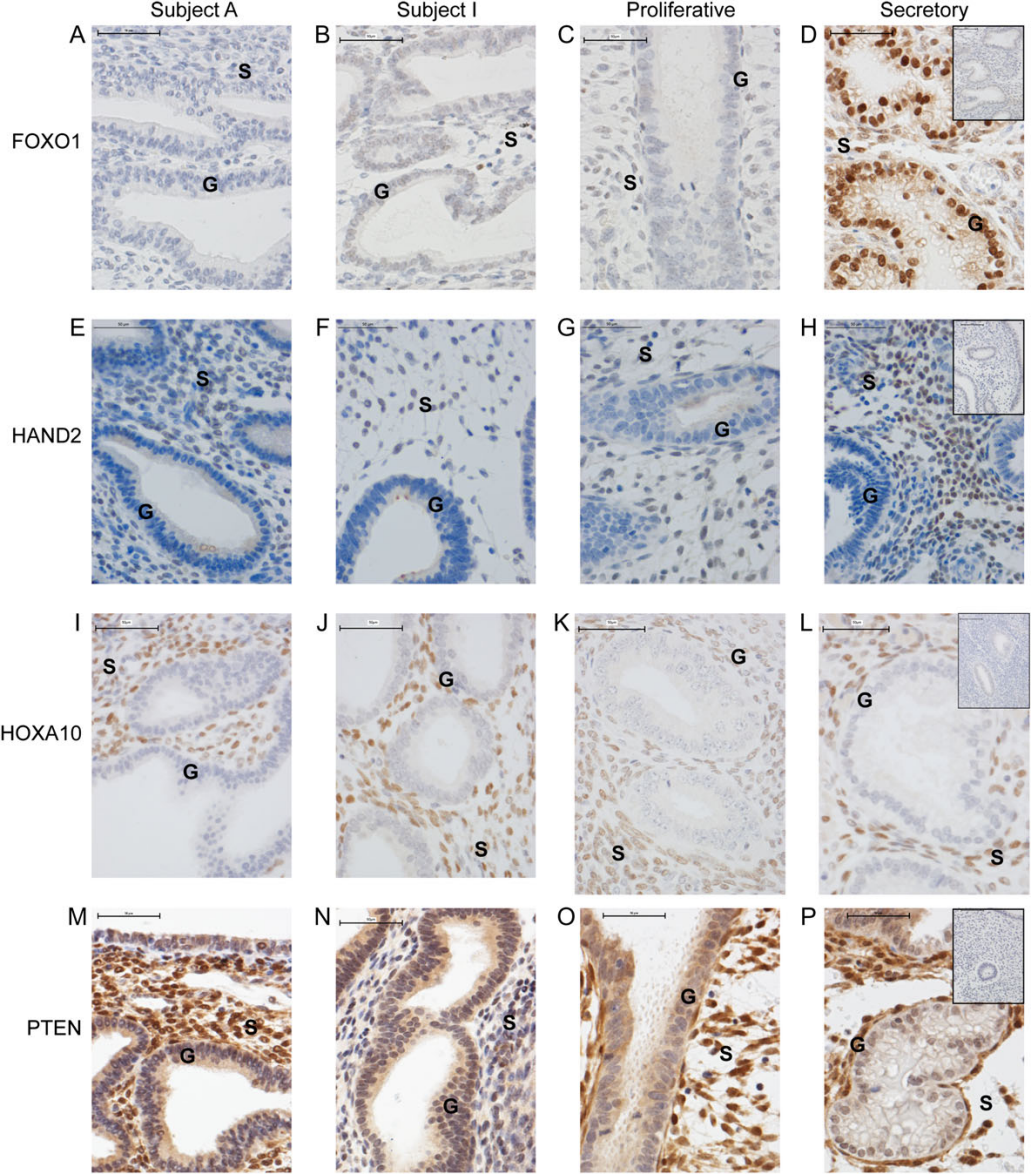
Effects of SPRM, UPA, administration on mRNA levels are reflected in modulation of protein localization. Representative images showing immuno-localization of FOXO1 (**A**–**D**), HAND2 (**E**–**H**), HOXA10 (**I**–**L**) and PTEN (**M**–**P**) in endometrium from woman with fibroids at proliferative and secretory stages and after UPA treatment. Subject A shows endometrium in which PAEC are characterized by extensive cystic glandular dilatation; Subject I has PAEC with minimal cystic glandular change. ×40 magnification (scale bar = 50 µm); G: Glands, S: Stroma. Negative controls shown as inserts on secretory endometrium.

In keeping with the impact of UPA on concentrations of mRNA; immunolocalisation of FOXO1 was intense in the nuclei of the glandular epithelium in secretory phase endometrium; immunostaining was minimal in both proliferative phase and following UPA treatment (Fig. 5A–D). HAND2 immunolocalisation was most dense in the nuclei of stromal fibroblasts of secretory phase endometrium with less intense staining in proliferative and UPA-treated endometrium (Fig. 5E–H). HOXA10 immunolocalisation was present in stromal cells of both control and UPA-treated samples, with less intense staining observed in proliferative phase compared with either secretory or UPA-treated samples (Fig. 5I–L). PTEN immunolocalisation paralleled that of mRNA concentrations with minimal alteration between groups (Fig. 5M–P).

### UPA administration does not increase endometrial cell proliferation

**Figure 6:**
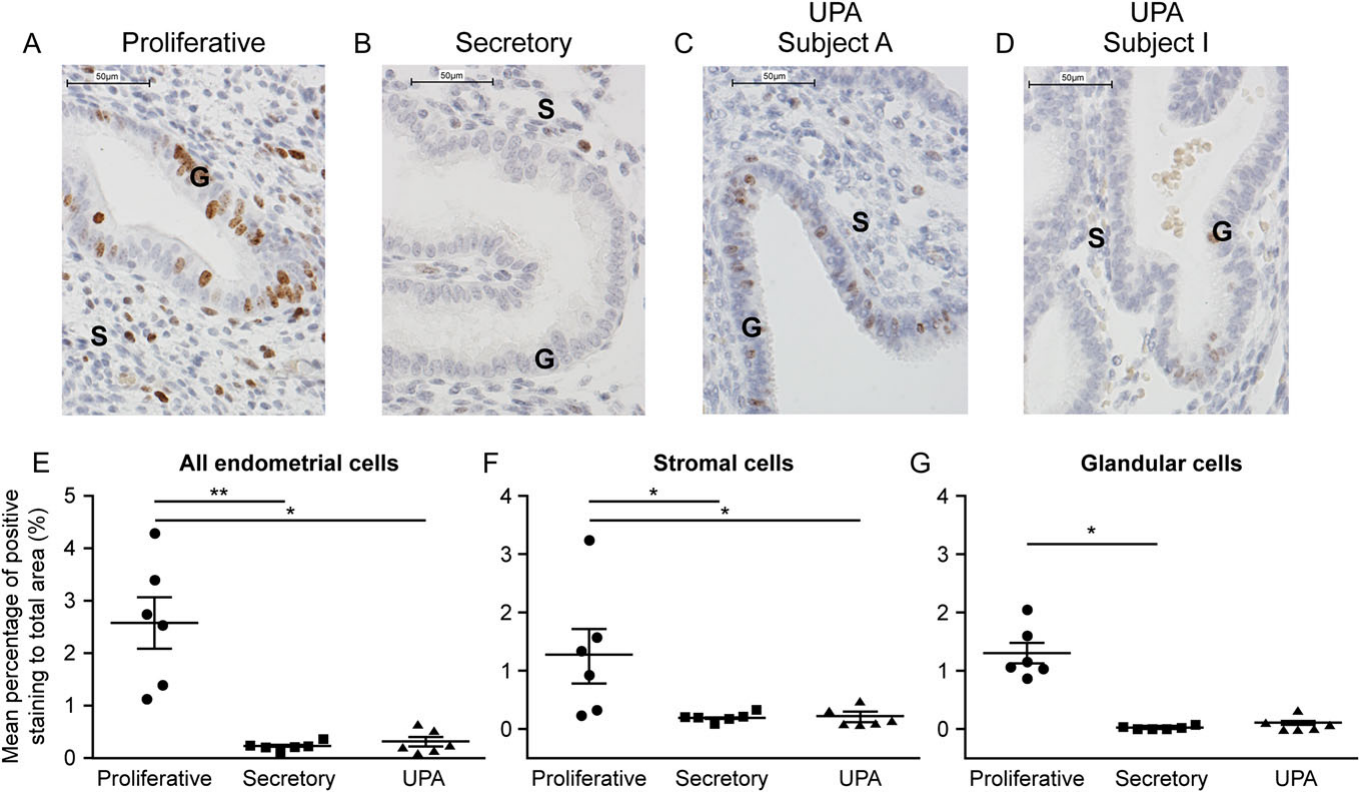
SPRM, UPA, administration does not increase endometrial cell proliferation. Proliferation assessed by Ki67 immunohistochemistry (**A**–**D**) and stereological quantification (**E**–**G**). Subject A shows endometrium in which PAEC are characterized by extensive cystic glandular dilatation; Subject I has PAEC with minimal cystic change. ×40 magnification (scale bar = 50 µm); G: Glands, S: Stroma. Kruskal–Wallis statistical test *P < 0.05; **P < 0.01.

Immunoexpression of Ki67 was used to assess cell proliferation within the stromal and glandular compartments of the endometrium. As expected immunopositive staining for Ki67 was detected in nuclei of both stromal cells and glandular epithelium in endometrium during proliferative phase and appeared more numerous than in secretory phase tissue (Fig. 6A and B). In glandular cells UPA treatment appeared to reduce Ki67 immunoexpression compared to samples from proliferative phase (Fig. 6C or D versus A). Quantification of proliferating cells confirmed that there were significantly more Ki67 positive cells in proliferative phase tissue compared to both secretory phase and UPA-treated endometrium (Fig. 6E). Analysis of stromal cells showed a significant reduction in Ki67 positive cells in secretory phase and in UPA-treated samples. In glandular epithelium Ki67 positive cells were significantly decreased in secretory compared to proliferative phase: UPA-treated tissue showed a similar trend although this did not reach significance (*P* = 0.069) (Fig. 6E–G).

## Discussion

UPA is a SPRM which, like other members of this class of compound, exhibits both agonist and antagonist activities *in vitro* (Wagenfeld *et al*., 2016). The impact of SPRMs is tissue-dependent and may be influenced both by bioavailabilty of different PR isoforms and the concentrations of different co-repressor and co-activator proteins in different cell types (Wagenfeld *et al*., 2016). In this study, we have demonstrated that UPA administration alters the pattern of expression of both PR and AR and mimics the anti-proliferative impact of progesterone in secretory phase endometrium.

We have conducted a comprehensive analysis of the pattern of gene expression in endometrium from women with fibroids treated with UPA prior to hysterectomy and compared this to untreated endometrium from women who also had fibroids. In keeping with the established literature (Williams *et al*., 2012) the extent of morphological change within the endometrium varied. Larger studies have demonstrated PAEC rates of around 60% following short but repeated courses of UPA administration although there is an evidence demonstrating that PAEC rapidly regresses on cessation of treatment (Donnez *et al*., 2014). Why not all subjects develop PAEC is uncertain and the impact of this morphological observation upon symptom control is unknown; in our study the degree of PAEC was not correlated with duration of treatment.

Two isoforms of PR are expressed in a differential manner within the endometrium (Mote *et al*., 1999). Our data herein demonstrate downregulation of both PR isoforms within the stroma and upregulation within the glandular epithelium following UPA administration. This finding is consistent with previous reports studying the effect of the SPRM asoprisnil on PR protein localization in human endometrium (Wilkens *et al*., 2013). The pattern of expression of *ESR1* (ERα) mRNA and protein after treatment with UPA was similar to that of the proliferative phase showing this SPRM did not induce PR-dependent downregulation of ERα gene expression. This finding is similar to findings for the PR-antagonist mifepristone when administered to Rhesus macaques (Slayden and Brenner, 1994).

One of the most striking and consistent findings in this study using the SPRM UPA was a significant increase in concentrations of *AR* mRNA, which was accompanied by a unique pattern of AR protein expression, distinct to that reported during the normal menstrual cycle (Marshall *et al*., 2011). These findings in human are consistent with reports from analysis of endometrium from Rhesus macaques following treatment with an intrauterine device containing UPA for three artificial menstrual cycles (Brenner *et al*., 2010). A similar effect has been reported following treatment with mifepristone, which increased stromal and glandular AR immunoexpression in Rhesus macaques (Slayden *et al*., 2001) and in women treated with a single post-ovulatory dose of the SPRM (Slayden *et al*., 2001).

To explore whether UPA was acting as an agonist or antagonist with respect to progesterone-dependent gene expression we measured mRNAs encoded by genes that have been identified in rodent studies where PR and PRB have been conditionally knocked-out or increased during the progesterone-dominated secretory phase of the cycle (Wetendorf and DeMayo, 2012). Consistent with previous reports, mRNAs encoded by *IGFBP-1, FOXO1, IL-15 and HAND2* were all significantly increased in secretory phase control samples compared with those in proliferative phase. Treatment with UPA resulted in mRNA concentrations similar to proliferative phase and significantly lower than secretory phase, consistent with lack of morphological differentiation and suggesting UPA exhibiting limited PR-dependent agonism in endometrium. Previous work using human endometrial stromal cells treated with a decidualisation stimulus has suggested that *HAND2* may regulate *FOXO1* and *IGFBP-1* expression (Huyen and Bany, 2011) and thus the reduction in *HAND2* by UPA may be implicated in the reduction of *FOXO1* and *IGFBP-1*. Other studies have shown expression of *FOXO1* in endometrial stromal cells is upregulated by cAMP and progesterone (Labied *et al*., 2006) and a genomic screen of human endometrial stromal cells treated with a decidualisation protocol showed 15% of the genes induced were aberrantly expressed if *FOXO1* was knocked down with key targets including *IGFBP-1* (Vasquez *et al*., 2015). As FOXO1 binding sites are present in the majority of DNA regions associated with PR binding (Vasquez *et al*., 2015) our finding of reduced expression of *FOXO1* in UPA-treated women may explain some of the changes in PR-dependent genes.

In the normal menstrual cycle IL-15 rises in response to progesterone (Dunn *et al*., 2002). Suppression of genes in the *IL-15* pathway have been reported following asoprisnil administration. This observation was corroborated with reduced expression of *IL-15* mRNA and the absence of endometrial CD-56 positive cells on immunolocalisation (Wilkens *et al*., 2013). In our current data set, *IL-15* expression in UPA-treated endometrium was reduced compared to secretory phase endometrium consistent with UPA lacking agonist activity; however, the impact upon the CD-56 positive immune-cell population was not investigated.

*IHH* has been identified as a rapidly induced mediator of PR activation that localizes to the luminal and glandular epithelium (Takamoto *et al*., 2002) and acts in a paracrine fashion to initiate a cascade of gene expression in the stromal cell compartment (Takamoto *et al*., 2002). Whilst one genome wide molecular phenotyping study indicated that *IHH* was downregulated during the progression of the endometrium through the secretory phase (Talbi *et al*., 2006), Wei *et al*. (2010) reported that IHH protein expression was upregulated in secretory when compared to proliferative endometrium. The same authors, also reported an upregulation of *IHH* mRNA of 12 women in response to UPA in proliferative endometrium suggesting that UPA administration may have had an agonistic PR effect. In our study, *IHH* mRNA was reduced in secretory phase samples compared with those in the proliferative phase. Treatment with UPA was associated with mRNA concentrations similar to those in the proliferative phase. There are a number of reasons that may explain these contrasting findings. The dose of UPA administered in our study was lower (5 mg daily) compared with 10–20 mg. In our study, all UPA patients exhibited PAEC to a greater or lesser degree in contrast to low levels of PAEC reported by Wei *et al*. It is unknown what impact the development of endometrial PAEC may have had on our data.

HOXA10 is an important transcription factor for endometrial development including decidualisation. HOXA10 is expressed in both glandular and stromal compartments and expression is regulated by both estradiol and progesterone (Eun Kwon and Taylor, 2004). In healthy women expression peaks during mid-secretory phase and its expression is considered an important factor in endometrial receptivity (Kulp *et al*., 2016). In women with uterine fibroids secretory phase upregulation may be impaired (Makker *et al*., 2016). As all our subjects had fibroids this may explain why we demonstrated a reduced expression of *HOXA10* mRNA from the secretory endometrium compared with proliferative phase at the PCR level although this was not reflected in protein expression. In those subjects exposed to UPA, *HOXA10* mRNA expression was similar to proliferative phase. Evidence regarding *HOXA10* gene expression in women with uterine pathology is beginning to emerge with evidence of alterations in gene methylation suggesting this may be a common molecular mechanism that becomes aberrant in some conditions (Kulp *et al*., 2016). Future studies may shed light on this by exploring the impact of UPA on *HOXA10* promoter methylation but this was outside the scope of the current study.

In this study, *COUP TFII* and *BMP2* mRNA levels were constant irrespective of stage of cycle or PR modulation or UPA treatment. PTEN acts as a tumour suppressor. Inactivation of this is a common feature of endometrial cancer, particularly endometrioid subtypes, and often predates morphological evidence of malignancy and pre-malignant precursors. Exogenous progestins may play an important role in elimination of PTEN-null glands (Orbo *et al*., 2006). Here we have shown that administration of UPA had little effect on PTEN, consistent with observations after asoprisnil administration (Wilkens *et al*., 2009).

In women treated with UPA circulating estrogen levels are maintained and it is therefore important to determine whether rates of endometrial cell proliferation increase with treatment. The Ki67 antigen is expressed during several phases of the cell cycle (G1, S, G2 and M), and is a well established prognostic and predictive biomarker and cell proliferation marker in clinical assessment of endometrium (Sivalingam *et al*., 2016). We thus considered Ki67 a reasonable choice for assessment of cell proliferation in endometrium in the present study.

In the current study, there was no evidence that rates of cell proliferation were increased compared with secretory phase and the number of ki67 immunopositive cells in both stromal and epithelial compartments was significantly lower than in proliferative phase. This is consistent with effects observed with other SPRMs (Wilkens *et al*., 2009) but such data have not previously been demonstrated in a quantitative manner following UPA administration. The mechanisms responsible for the low level of proliferation in UPA-treated women have not been explored. AR regulation has been implicated in the endometrial anti-proliferative effect observed with mifepristone administration (Slayden and Brenner, 1994). It is notable that AR is present in both stromal and epithelial cell nuclei and androgens are known to have anti-proliferative effects on endometrial tissue (Miller *et al*., 1986). In primates co-administration of mifepristone with the anti-androgen flutamide abolished the reduction in mitotic indices and endometrial atrophy (Brenner *et al*., 2003). The overexpression of AR in epithelial cells may be implicated in the low epithelial cell proliferation after UPA treatment. The impact of androgens on development of endometrial malignancy is complex (Gibson *et al*., 2014) and further studies on the relationship between UPA and AR are merited. UPA-dependent changes in expression of other factors may also play a role in the low levels of cell proliferation. For example, mRNAs encoded by *FOXM1*, a transcription factor that plays crucial roles in cell proliferation (Gao *et al*., 2015), were highest in the proliferative phase samples consistent with previous reports. *FOXM1* was reduced in both secretory phase and UPA-treated samples and whether the low level of this transcription factor contributes to reduced cell proliferation merits further investigation.

## Conclusions

New medical therapies for HMB are required and UPA has shown promise as a treatment for fibroids but its direct impacts on endometrium have received less attention. In this study, we have focused on an in-depth analysis of a cohort of women treated with UPA for 9–12 weeks prior to hysterectomy. The treatment resulted in changes in endometrial morphology and gene expression consistent with UPA acting as a PR antagonist in the absence of an increased rate of endometrial cell proliferation. These novel data indicate that UPA has the potential to fill an unmet clinical need in treatment of HMB.
